# *N*-Methyl-d-aspartic Acid (NMDA) Receptor Is Involved in the Inhibitory Effect of Ketamine on Human Sperm Functions

**DOI:** 10.3390/ijms222212370

**Published:** 2021-11-16

**Authors:** Ying Chen, Wenqing Xu, Yuan Yuan, Houyang Chen, Shuangyan Zheng, Yuanqiao He, Tao Luo

**Affiliations:** 1 Institute of Life Science and School of Life Science, Nanchang University, Nanchang 330031, China; chenying26@ncu.edu.cn (Y.C.); ncuskxuwenqing@163.com (W.X.); yuanyuanbling@163.com (Y.Y.); 2 Key Laboratory of Reproductive Physiology and Pathology in Jiangxi Province, Nanchang 330031, China; chenhouyang2007@163.com; 3 Department of Laboratory Animal Science, Nanchang University, Nanchang 330031, China; zsyan59@163.com; 4 Jiangxi Province Key Laboratory of Laboratory Animal, Nanchang 330031, China

**Keywords:** NMDA receptor, NMDA, ketamine, human sperm functions, intracellular calcium concentration

## Abstract

Ketamine, which used to be widely applied in human and animal medicine as a dissociative anesthetic, has become a popular recreational drug because of its hallucinogenic effect. Our previous study preliminarily proved that ketamine could inhibit human sperm function by affecting intracellular calcium concentration ([Ca^2+^]_i_). However, the specific signaling pathway of [Ca^2+^]_i_ induced by ketamine in human sperm is still not clear yet. Here, the *N*-methyl-d-aspartic acid (NMDA) receptor was detected in the tail region of human sperm. Its physiological ligand, NMDA (50 μM), could reverse ketamine’s inhibitory effect on human sperm function, and its antagonist, MK801 (100 μM), could restrain the effect of NMDA. The inhibitory effect caused by 4 mM ketamine or 100 μM MK801 on [Ca^2+^]_i_, which is a central factor in the regulation of human sperm function, could also be recovered by 50 μM NMDA. The results suggest that the NMDA receptor is probably involved in the inhibitory effect of ketamine on human sperm functions.

## 1. Introduction

Ketamine used to be widely applied in human and veterinary medicine as a dissociative anesthetic. However, ketamine has gradually become a popular recreational drug in entertainment venues because of its hallucinogenic effect [1]. The toxic effects of ketamine on human reproduction have attracted extensive attention because of the expansion of the ketamine abuse population and a worldwide decrease in male fertility. A previous study preliminarily demonstrated that ketamine could inhibit human sperm function by reducing intracellular calcium concentration ([Ca^2+^]_i_) [2]. However, the specific signaling pathway of [Ca^2+^]_i_ induced by ketamine in human sperm is still unknown.

Ketamine serves as a low-affinity, use-dependent, noncompetitive antagonist of glutamatergic *N*-methyl-d-aspartate (NMDA) receptor. It can block NMDA receptors to impact the normal physiological function of cells, especially in neurons [3]. It has been reported that ketamine inhibits the NMDA receptor by two distinct mechanisms. It either blocks the open channel by reducing the mean channel open time or decreases the frequency of opening for the closed channel by an allosteric mechanism [4]. Interestingly, mature human sperm is vividly referred to neurons with tails because of its expression of some neurotransmitter receptors, such as gamma-aminobutyric acid (GABA) and glycine receptors [5]. Although the NMDA receptor is a ligand-gated cation channel that causes Ca^2+^ influx in neurons, it also has functions in the reproductive system of various species. For example, Endo et al. proved that the NMDA receptor mediates the acrosome reaction and motility initiation in *newt* sperm [6]. In vertebrates, NMDA receptors in Leydig cells and spermatogonia are involved in the regulation of steroidogenesis and spermatogenesis in the testis [7]. Froman demonstrated that seminal plasma glutamate acts as a motility agonist via the NMDA receptor to fuel the sperm stored in the *fowl* oviduct to ascend the vagina [8]. However, whether NMDA receptor is expressed and has functions in mature human sperm is still unclear. Considering that the NMDA receptor is the main target of ketamine, studying these issues will contribute to further elucidating the mechanism of ketamine in the function of mature human sperm.

In this study, the expression of the NMDA receptor was identified by immunofluorescence. Whether the receptor is involved in the regulation of the human sperm function by ketamine was further studied by means of a sperm function experiment and intracellular calcium evaluation. Our study is expected to clarify the regulatory mechanism of ketamine for human sperm.

## 2. Results

### 2.1. NMDA Receptor Was Expressed in Human Sperm

**Figure 1:**
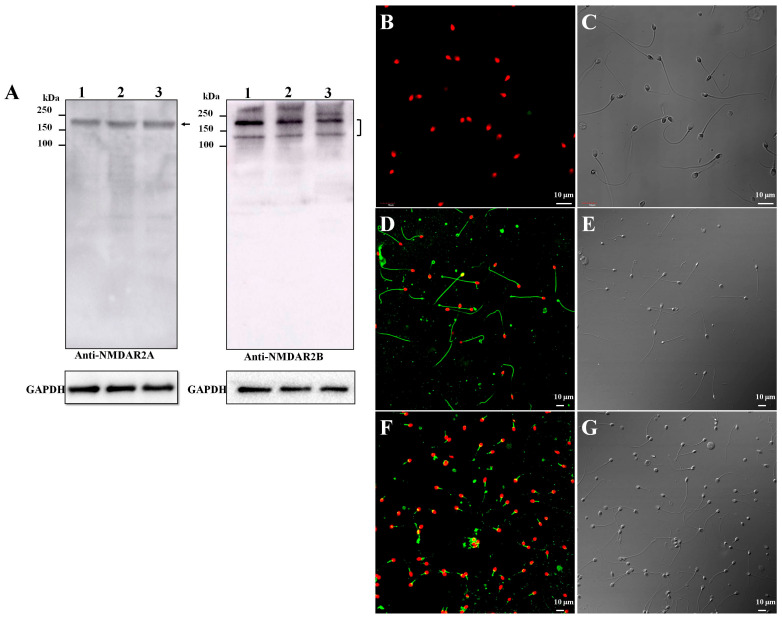
Expression of two NMDA receptor subunits, NMDAR2A and NMDAR2B, in human sperm. (**A**) The antibodies, Western blotting. GAPDH was blotted as the negative control. (**B**,**C**) Rabbit IgG was taken as the negative control. (**D**) Immunofluorescence staining and (**E**) bright-field microscopy of NMDAR2A. (**F**) Immunofluorescence staining and (**G**) bright-field microscopy of NMDAR2B.

Considering that the NMDA receptor is the main target of ketamine, detecting the expression of the NMDA receptor in human sperm is necessary. The Human Protein Atlas database analyses revealed the existence of five NMDA receptor subunits in humans, namely, NMDAR1, NMDAR2A, NMDAR2B, NMDAR2C, and NMDAR2D. Among them, NMDAR2A and NMDAR2B had the highest expression in the testis. Moreover, NMDAR2A and NMDAR2B are the most abundant in mammals [9]. Therefore, Western blot analysis was first performed on proteins from freshly collected human sperm to further confirm the expression of the NMDA receptor in human sperm. The target band between 150 and 250 kDa (the predicted sizes of NMDAR2A and NMDAR2B were 165 and 166 kDa, respectively) could be obviously observed (Figure 1A). Moreover, immunolocalization was also performed on freshly collected sperm by confocal microscopic analysis to visualize the expression pattern of NMDAR2A and NMDARN2B in human sperm. The immunofluorescence results showed that the two NMDA receptor subtypes were expressed in human sperm. NMDAR2A was located in the entire sperm tail (Figure 1D,E), whereas NMDAR2B was concentrated in the middle piece of the sperm tail (Figure 1F,G). The rabbit IgG was taken as the negative control (Figure 1B,C). These results support the endogenous expression of the receptor in human sperm.

### 2.2. NMDA Could Alleviate the Inhibitory Effect of Ketamine on Human Sperm Motility

**Figure 2:**
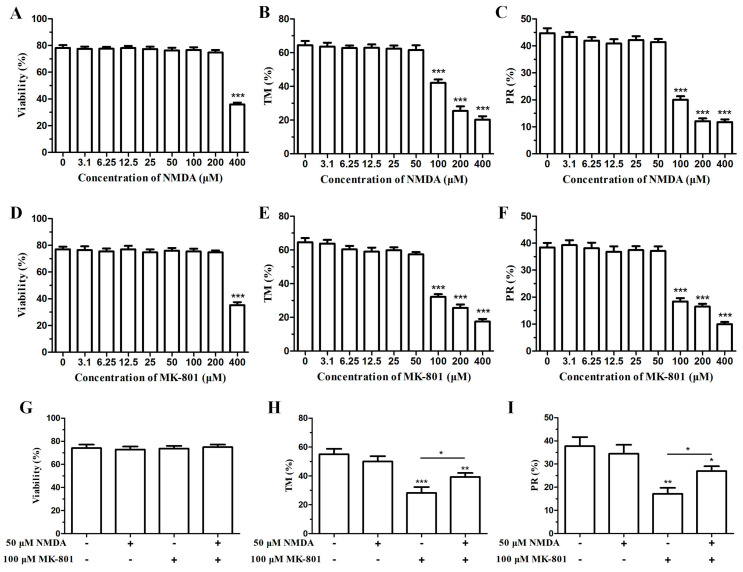
Effects of the NMDA receptor agonist NMDA and antagonist MK-801 on human sperm motility. Effects of different NMDA concentrations on the viability (**A**), total motility (TM, **B**), and progressive motility (PR, **C**) of human sperm, respectively (*n* = 8). Effects of different MK-801 concentrations on the viability (**D**), total motility (**E**), and progressive motility (**F**) of human sperm (*n* = 8). (**G**) Effect of the separate application or combination of 50 μM NMDA and 100 μM MK-801 on human sperm viability (*n* = 8). (**H**,**I**) Effects of the separate application or combination of 50 μM NMDA and 100 μM MK-801 on the total motility and progressive motility of human sperm (*n* = 6). Bar: mean ± SEM, one-way ANOVA; * *p* < 0.05, ** *p* < 0.01, *** *p* < 0.001.

NMDA, the physiological ligand of the NMDA receptor, was utilized to detect its effect on ketamine’s ability to inhibit human sperm motility and verify whether the NMDA receptor is involved in the effect of ketamine on human sperm. First, the cytotoxicity of NMDA to human sperm was evaluated. NMDA (<400 μM) did not affect sperm viability (Figure 2A). Additionally, 3.1–50 μM NMDA had no effect on the total motility and progressive motility of human sperm (Figure 2B,C). The results showed that the most suitable NMDA concentration was 50 μM because it has no impact on the viability and motility of human sperm.

**Figure 3:**
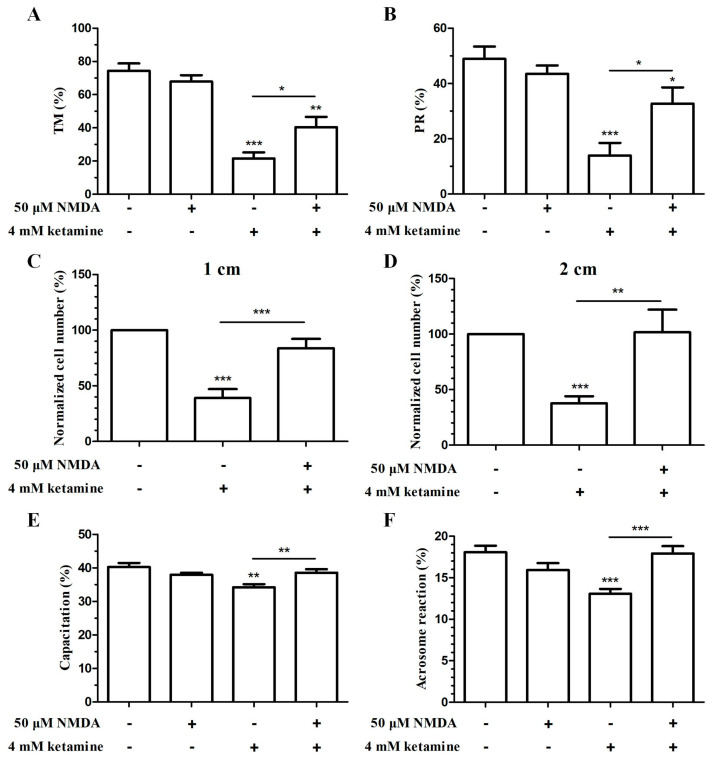
The NMDA receptor participated in ketamine inhibitory effects on human sperm functions, including motility, penetration ability, capacitation, and acrosome reaction. Effects of 4 mM ketamine and 4 mM ketamine plus 50 μM NMDA on human sperm total motility (**A**) and progressive motility (**B**) (*n* = 7). (**C**,**D**) Effects of 4 mM ketamine and 4 mM ketamine plus 50 μM NMDA on human sperm penetration ability (*n* = 15). Human sperm was incubated with 4 mM ketamine and 4 mM ketamine plus 50 μM NMDA in HTF++ at 37 °C in an incubator with 5% CO_2_ for 4 h. The capacitation (**E**) and acrosome reaction (**F**) of human spermatozoa were examined by CTC staining. Two hundred sperms were counted in each assay to evaluate the different CTC staining patterns. Bar: mean ± SEM, *n* = 7, one-way ANOVA; * *p* < 0.05, ** *p* < 0.01, *** *p* < 0.001.

The total motility and progressive motility of sperm remarkably decreased after treatment with 4 mM ketamine compared with the control group (Figure 3A,B). Meanwhile, the inhibitory effect of ketamine on sperm motility could be recovered substantially by preincubation with 4 mM ketamine mixed with 50 μM NMDA, although the total motility and progressive motility were also decreased compared with the control group (Figure 3A,B).

In addition, the ability of sperm to penetrate the 1% (*w*/*v*) methylcellulose solution, which imitates the movement process of the female reproductive tract, was also measured. The results showed that although 50 μM NMDA had no remarkable effect on the penetration ability of human sperm (Figure S1), it could almost completely reverse the ketamine-induced inhibition of the penetration ability of human sperm (Figure 3C,D). The above results indicated that the inhibitory effect of ketamine on the motility of human sperm could be partly alleviated by 50 μM NMDA.

### 2.3. NMDA Could Remarkably Reverse the Inhibitory Effect of Ketamine on the Capacitation and Acrosome Reaction of Human Sperm

Before sperm–egg binding and fusion, the sperm must undergo the acrosome reaction to dissolve the zona pellucid [10,11], and capacitation is the prerequisite for the acrosome reaction [12]. Therefore, the effects of ketamine and the NMDA–ketamine mixture on the two vital processes in human sperm fertilization were measured. The results showed that 4 mM ketamine could remarkably reduce the capacitation and acrosome reaction rate, both of which could be significantly recovered by 50 μM NMDA (Figure 3E,F).

### 2.4. NMDA Receptor Antagonist MK-801 Could Reduce Human Sperm Motility

MK-801, a potent, selective, and noncompetitive specific antagonist of the NMDA receptor which acts by binding to a site in NMDA-related ion channels to prevent the [Ca^2+^]_i_ flow [13,14], was utilized to confirm whether the NMDA receptor is associated with the inhibiting effect of ketamine on human sperm function. The cytotoxicity of MK-801 on human sperm was first evaluated. The results showed that <400 μM MK-801 do not affect sperm viability (Figure 2D). Moreover, 100 μM MK-801 remarkably suppressed the total motility and progressive motility of human sperm (Figure 2E,F). In comparison, the combination of 100 μM MK-801 and 50 μM NMDA did not affect human sperm viability (Figure 2G). In addition, similar to the inhibitory effect of ketamine, the depression of the total motility and progressive motility of human sperm caused by MK-801 could be partly reversed by 50 μM NMDA (Figure 2H,I). These results indicated that the NMDA receptor probably participates in the motion of human sperm.

### 2.5. NMDA Could Recover the Inhibitory Effect of Ketamine on [Ca^2+^]_i_ of Human Sperm

**Figure 4:**
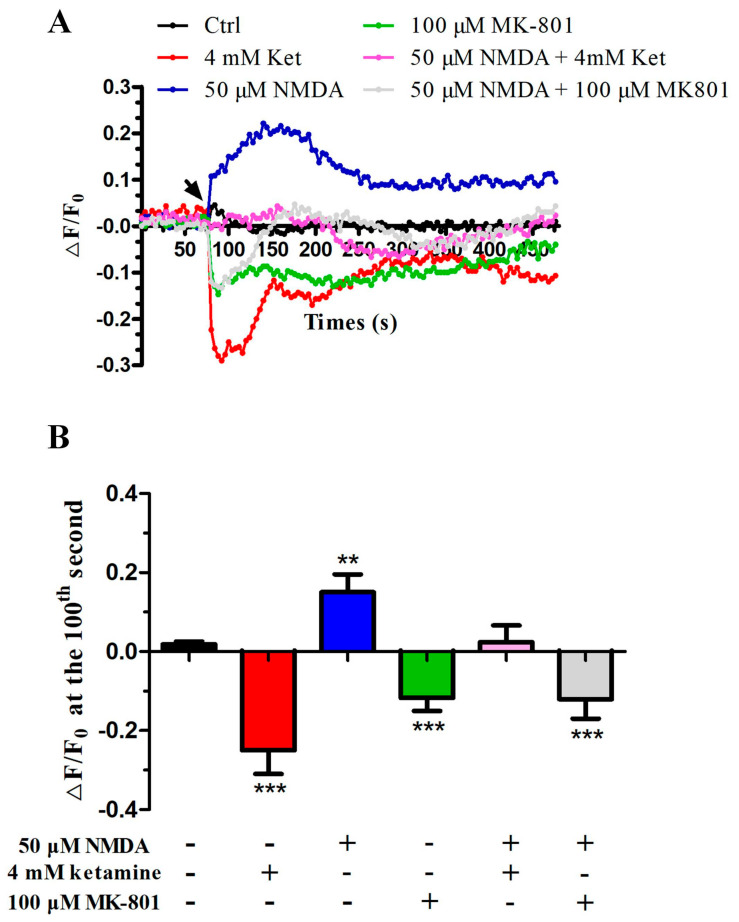
The NMDA receptor involved in ketamine suppression effects on the intracellular calcium concentration of human sperm. (**A**) Time course curve of real-time changes in human sperm [Ca^2+^]_i_. The arrow indicates the time when ketamine (4 mM), NMDA (50 μM), MK801 (100 μM), and NMDA (50 μM) plus ketamine (4 mM)/MK801 (100 μM) were added into the sperm samples. (**B**) Inhibition effects of different chemicals on human sperm [Ca^2+^]_i_ at the 100th second as calculated by Δ*F/F*_0_ from the time course curve through statistical analysis. Bar: mean ± SEM, *n* = 7, one-way ANOVA; ** *p* < 0.01, *** *p* < 0.001.

The previous results in this study indicated that ketamine significantly restrained sperm motility, penetrated ability, capacitation, and acrosome reaction of human sperm, all of which could be recovered by NMDA. These physiological processes during sperm fertilization were closely related to the change in the [Ca^2+^]_i_ level. Thus, real-time changes in human sperm [Ca^2+^]_i_ were monitored in a fluorescence plate reader after sperm was loaded and stained with Fluo-4 AM to verify our hypothesis. The results showed that 4 mM ketamine and 100 μM MK-801 could decrease human sperm [Ca^2+^]_i_ by 25% and 15%, respectively, in a very short time, whereas 50 μM NMDA could increase [Ca^2+^]_i_ alone by 20% or recover the inhibitory effect of ketamine or MK-801 to the basal level (Figure 4).

## 3. Discussion

Ketamine has toxic effects on the male reproductive system of humans and animals [15,16,17]. Ketamine exposure in vitro has toxic effects on human sperm functions, including viability, motility, penetration ability, capacitation, and acrosome reaction, and reduces [Ca^2+^]_i_ [2]. However, the intracellular signaling pathway of ketamine toxicity to human sperm function is still unknown. Considering that the population of ketamine abusers is growing, the effect of ketamine on sperm function and its mechanism is particularly important to clarify. In this study, we authenticated the expression of the NMDA receptor in the tail region of human sperm and proved that NMDA, the physiological ligand of the receptor, could reverse the inhibitory effects of ketamine on sperm functions, including motility, penetration ability, capacitation, and acrosome reaction, as well as [Ca^2+^]_i_. The results indicated that NMDA and ketamine may compete for the binding site of the NMDA receptor, and the NMDA receptor is probably involved in the inhibitory effect of ketamine on human sperm function.

Mature mammalian sperm chromosomes are highly condensed, and the transcription is thought to be silent [18]. Therefore, post-transcriptional processes, especially [Ca^2+^]_i_, play a vital role in sperm function regulation [19,20]. The change in [Ca^2+^]_i_ in mammalian sperm can be achieved by the Ca^2+^ flux into the cytoplasm from the extracellular fluid or by Ca^2+^ release from calcium stores within the cell [21,22]. As one of important ligand-gated cation channels that induce the Ca^2+^ influx in neurons, the NMDA receptor is expressed and functions in mouse sperm [23]; thus, it is involved in the initiation of the acrosome reaction [24]. In this study, ketamine and MK-801 (NMDA receptor antagonist) depressed [Ca^2+^]_i_ and sperm function, whereas NMDA (physiological agonist of the NMDA receptor) could reverse this inhibitory effect; thus, the NMDA receptor probably participates in intracellular calcium regulation in sperm and further impacts sperm function. Interestingly, a number of plasma membrane receptor types, such as NMDA receptors, GABA receptors, and glycine receptors, which were originally thought to be specific to neurons, have been found in sperm [5]. In mammals, sperm and neurons share similar functions, that is, they activate other cells (other neurons or somatic effector cells by neurons or the egg by a sperm). However, the activation mechanisms involved are quite different; “cell-to-cell” contact is required in the case of sperm, whereas chemical contact (chemical synapses) or gap junctions are required in the case of neurons. Although the specific functions of the sperm and neurons are remarkably different, both require exocytosis to carry out their function [10,25]. Several “neuronal” receptors present in sperm appear to be involved in the direct control of the acrosome reaction [5], which is an exocytotic event essential to fertilization. In this study, we also demonstrated that NMDA could recover the inhibitory effect of ketamine on the acrosome reaction of human sperm. Hence, considering that the NMDA receptor is involved in sperm function, the natural ligands of the receptor, such as NMDA, glutamic acid, and aspartic acid as agonists and Mg^2+^ and Zn^2+^ as antagonists, should be highly concerned in clinical studies, as the types and concentrations of these ligands in the semen are likely to be related to sperm function. Besides, other “neuronal” receptors in human sperm should also be further studied with regard to this issue.

In conclusion, the NMDA receptor was detected by immunofluorescence and its location was mainly focused on the tail region. In addition, the inhibitory effects of in vitro exposure to 4 mM ketamine or 100 μM MK801 of human sperm function, including motility, penetration ability, capacitation, and acrosome reaction, could be reversed by 50 μM NMDA, the physiological ligand of the NMDA receptor. Furthermore, sperm [Ca^2+^]_i_ could also be restored from the suppression of ketamine or MK801. Our findings revealed that the NMDA receptor was probably involved in the ketamine toxicity of human sperm function and provided a reference for clinical diagnosis of ketamine abusers.

## 4. Materials and Methods

### 4.1. Human Sperm Sample Collection and Preparation

The collection of human semen samples and experiments in this study were approved by the Institutional Ethics Committee on Human Subjects of The Second Affiliated Hospital of Nanchang University. Semen samples were collected by masturbation after 3–5 days of sexual abstinence from 25 normozoospermic donors aged 23–32 with known reproductive histories in the past 2 years and normal testicular and semen parameters according to the World Health Organization (WHO, Geneva, Switzerland) laboratory manual for the examination and processing of human semen. The donors who participated in this study signed informed consent forms. Semen samples were liquefied for 30 min after fresh collection and relevant experiments were immediately performed. Sperms were harvested by direct swim-up, if necessary, in a bath solution (HS) containing 135 mM NaCl, 5 mM KCl, 1 mM MgSO_4_, 2 mM CaCl_2_, 5 mM glucose, 20 mM 4-(2-hydroxyethyl)-1-piperazineethanesulfonic acid (HEPES), 1 mM Na pyruvate, and 10 mM lactic acid at pH 7.4 adjusted with NaOH.

### 4.2. Measurement of Sperm Motility and Viability

Two sperm motion parameters (i.e., total motility (TM) and progressive motility (PR)) were assessed by Hamilton Thorne CEROSII computer-aided sperm analysis (Hamilton Thorne Biosciences, Beverly, MA, USA) according to the WHO laboratory manual for the examination and processing of human semen, and a minimum of 200 sperms were counted for each assay. Briefly, after swim-up, the sperm was resuspended in the HS medium incubated with the relevant chemicals for 30 min, and then sperm motility was measured. The sperm viability was evaluated by hematoxylin and eosin (HE) staining. The stained sperm was defined as dead while viable sperms were not stained. The percentage of viable sperm in each group was calculated as sperm viability.

### 4.3. Penetration of the Artificial Viscous Medium

Penetration ability was analyzed by evaluating sperm penetration of the 1% (*w*/*v*) methylcellulose solution prepared in HTF++ (HTF (human tubal fluid) plus 25 mM NaHCO_3_ and 0.4% human serum albumin (HSA; Vitrolife, Göteborg, Sweden); HTF: 93.8 mM NaCl, 4.69 mM KCl, 0.2 mM MgSO_4_, 0.37 mM KH_2_PO_4_, 2.04 mM CaCl_2_, 21.4 mM lactic acid, 2.78 mM glucose, 21 mM HEPES, and 0.33 mM Na pyruvate at pH 7.35 adjusted with NaOH) as previously described [2]. The methylcellulose was introduced into 7.5 cm flattened capillary tubes with 1.0 mm inner depth (Elite Medical Co., Ltd., Nanjing, China). Then, one end was sealed with plasticine. Before the penetration, sperm was suspended in the HTF++ medium for 3 h at 37 °C in a 5% CO_2_ incubator. After the relevant chemicals were added to the capacitated sperm samples, the open ends of the capillary tubes were inserted and the penetration started. After 1 h, the tubes were removed, wiped, and observed under a microscope. Three fields at 1 and 2 cm from the base of the tube were counted, respectively, and the average number of cells per field was calculated.

### 4.4. Evaluation of Capacitation and Acrosome Reaction

In brief, in order to determine the effect of the chemicals on sperm capacitation and spontaneous acrosome reaction, the sperm was incubated in the HTF++ medium containing the relevant chemicals for 4 h at 37 °C in a 5% CO_2_ incubator. The capacitation and acrosome reaction of human sperm were detected using the chlortetracycline (CTC) staining method, and a total of 200 sperms were counted to assess different CTC staining patterns as follows: “F”, fluorescence over the entire sperm head, intact acrosome, incapacitated sperm; “B”, a fluorescence-free band in the post-acrosomal region, intact acrosome, capacitated sperm; “AR,” fluorescence absent from the sperm head, acrosome-reacted sperm. The capacitated sperms were quantified as the sum of “B” and “AR”. Sperm suspension incubated in the HTF++ medium without chemical treatment was taken as the control group.

### 4.5. Measurement of Sperm [Ca^2+^]_i_

Sperm was loaded with 5 μM Fluo-4 AM and 0.05% Pluronic F-127 for 30 min at room temperature in the dark and subsequently washed in HS. The washed sperm was loaded on a microplate (Thermo Fisher Scientific, Waltham, MA, USA), and the Fluo-4 fluorescent was detected by an EnSpire^®^ multimode plate reader. The sperm was recorded for 80 s before the addition of ketamine (or NMDA or other chemicals) in HS and for 400 s after the administration of chemicals. The change in sperm [Ca^2+^]_i_ was calculated by Δ*F/F_0_* (%), which indicates the percentage (%) of fluorescence changes (Δ*F*) normalized to the mean basal fluorescence before the application of any chemicals (*F*_0_). Δ*F/F*_0_ (%) = (*F* − *F*_0_)/*F*_0_ × 100%, where F indicates the fluorescent intensity at each recorded timepoint (2 s per timepoint).

### 4.6. Western Blot Analysis

Total sperm protein was isolated, and protein concentration was determined using the bicinchoninic acid method. About 15 μg protein was electrophoresed on the 8% sodium dodecyl sulfate–polyacrylamide gel for 90 min and transferred onto polyvinylidene difluoride membranes (GE Healthcare, Pittsburgh, PA, USA). The protein was incubated with anti-NMDAR2A/NMDAR2B (Abcam, Cambridge, UK; 1:1000) as the primary antibodies and horseradish peroxidase-conjugated anti-rabbit IgG (CWBIO, Beijing, China; 1:5000) as the secondary antibody at room temperature for 60 min. The membranes were then visualized using an enhanced chemiluminescence detection kit (Proteintech, Wuhan, China) under a Bio-Rad gel imaging system (Bio-Rad, Hercules, CA, USA). GAPDH was blotted as the negative control. The experiments were repeated at least three times.

### 4.7. Immunofluorescence Analysis

Fresh human sperm was collected and fixed with 4% paraformaldehyde (Solarbio, Beijing, China) for 10 min. The fixed sperm was applied to the bottom of a confocal dish (Nest Biotechnology Co., Ltd., Wuxi, China) pretreated with 1% polylysine (Solarbio, Beijing, China) for 20 min. Afterwards, the sperm was permeabilized with 0.2% Triton X-100 (Solarbio, Beijing, China) in PBS (Solarbio, Beijing, China) for 15 min and subsequently blocked with 10% normal goat serum (NGS; Solarbio, Beijing, China) containing 0.05% Triton X-100 in PBS for at least 90 min at room temperature. Anti-GRIN2A/GRIN2B antibodies (Abcam, USA; 1:100) were incubated with 5% NGS containing 0.05% Triton X-100 in PBS overnight at 4 °C. DyLight 488-labeled goat anti rabbit IgG (EarthOx, San Francisco, CA, USA; 1:100) was used as the secondary antibody. The rabbit IgG (Solarbio, Beijing, China; 1:200) was taken as the negative control. All the images were obtained under a confocal laser scanning microscope (FV1000, Olympus, Tokyo, Japan) with an IX81 objective and analyzed with FV10-ASW 1.7 Viewer and Adobe Photoshop. The experiments were repeated at least three times.

### 4.8. Statistical Analysis

The data are expressed as the means ± standard error of the mean (SEM) and follow normal distribution as measured by the Shapiro–Wilk test (*p* > 0.05). Differences between the control and each treatment with chemicals were assessed by one-way ANOVA and Dunnett’s test using the GraphPad Prism version 6.0 software (San Diego, CA, USA). Differences with *p* < 0.05 were considered statistically significant.

## Supplementary Materials

The following are available online at https://www.mdpi.com/article/10.3390/ijms222212370/s1.
