# Supplementary material for: N-Methyl-d-aspartic Acid (NMDA) Receptor Is Involved in the Inhibitory Effect of Ketamine on Human Sperm Functions
Source: Int J Mol Sci. 2021 Nov 16;22(22):12370. doi: 10.3390/ijms222212370 (PMC8622018; doi:10.3390/ijms222212370)
Supplement: Supplementary file 1 [file ijms-22-12370-s001.zip › ijms-1416343-supplementary.pdf]

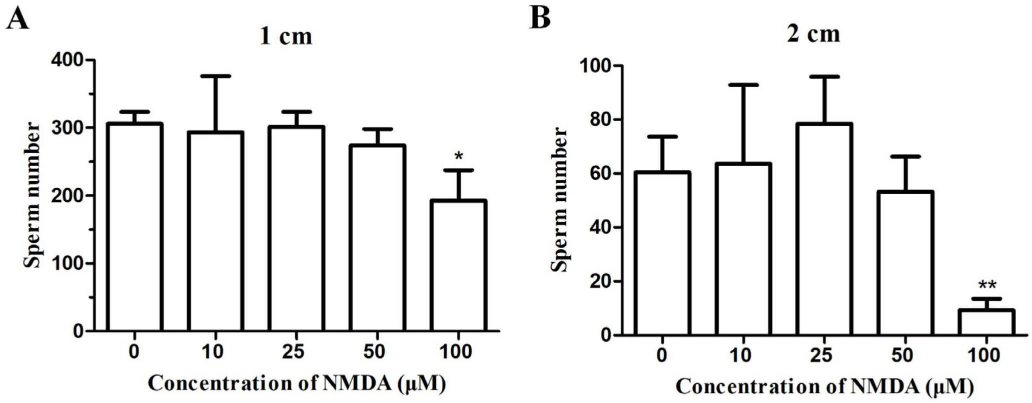

Supplementary Figure S1. Effect of NMDA on the penetration ability of human sperm. The effect of 10–100 μM NMDA on human sperm penetration ability. n = 9. Bar: mean ± SEM, one-way ANOVA. * $p < 0.05$, ** $p < 0.01$.
